# Evaluation of differences in expression pattern of three isoforms of the transforming growth factor beta in patients with lumbosacral stenosis

**DOI:** 10.1080/15384101.2024.2345484

**Published:** 2024-05-02

**Authors:** Dawid Sobański, Paweł Bogdał, Rafał Staszkiewicz, Małgorzata Sobańska, Michał Filipowicz, Ryszard Adam Czepko, Damian Strojny, Beniamin Oskar Grabarek

**Affiliations:** a Department of Neurosurgery, Szpital sw. Rafala in Cracow, Cracow, Poland; b Collegium Medicum, WSB University, Dabrowa Gornicza, Poland; c Department of Orthopedic, Szpital Powiatowy w Zawierciu, Zawiercie, Poland; d Department of Neurosurgery, 5th Military Clinical Hospital with the SP ZOZ Polyclinic in Krakow, Krakow, Poland; e Department of Neurosurgery, Faculty of Medicine in Zabrze, Academy of Silesia, Katowice, Poland; f Department of Neurosurgery, Faculty of Medicine and Health Sciences, Andrzej Frycz Modrzewski University in Cracow, Cracow, Poland; g Institute of Health Care, National Academy of Applied Sciences in Przemyśl, Przemyśl, Poland; h Department of Medical Science, New Medical Techniques Specialist Hospital of St. Family in Rudna Mała, Rzeszów, Poland

**Keywords:** Yellow ligamentum flavum, degenerative spinal stenosis, transforming growth factor beta

## Abstract

The study investigates molecular changes in the lumbosacral (L/S) spine’s yellow ligamentum flavum during degenerative stenosis, focusing on the role of transforming growth factor beta 1–3 (TGF-β-1–3). Sixty patients with degenerative stenosis and sixty control participants underwent molecular analysis using real-time quantitative reverse transcription reaction technique (RTqPCR), enzyme-linked immunosorbent assay (ELISA), Western blot, and immunohistochemical analysis (IHC). At the mRNA level, study samples showed reduced expression of *TGF-β-1* and *TGF-β-3*, while *TGF-β-2* increased by only 4%. Conversely, at the protein level, the study group exhibited significantly higher concentrations of TGF-β-1, TGF-β-2, and TGF-β-3 compared to controls. On the other hand, at the protein level, a statistically significant higher concentration of TGF-β-1 was observed (2139.33 pg/mL ± 2593.72 pg/mL vs. 252.45 pg/mL ± 83.89 pg/mL; *p* < 0.0001), TGF-β-2 (3104.34 pg/mL ± 1192.74 pg/mL vs. 258.86 pg/mL ± 82.98 pg/mL; *p* < 0.0001), TGF-β-3 (512.75 pg/mL ± 107.36 pg/mL vs. 55.06 pg/mL ± 9.83 pg/mL, *p* < 0.0001) in yellow ligaments obtained from patients of the study group compared to control samples. The study did not establish a significant correlation between TGF-β-1–3 concentrations and pain severity. The findings suggest that molecular therapy aimed at restoring the normal expression pattern of TGF-β-1–3 could be a promising strategy for treating degenerative stenosis of the L/S spine. The study underscores the potential therapeutic significance of addressing molecular changes at the TGF-β isoforms level for better understanding and managing degenerative spinal conditions.

## Introduction

The lumbosacral (L/S) section of the spine is a structure that provides mobility between the pelvis and chest, as well as being the attachment site for some abdominal and back muscles. Throughout a person’s life, the L/S section of the spine is exposed to internal forces, which include the forces of muscle tension and external forces, i.e. gravity and forces arising from the action of external factors [1–3].

L/S spinal stenosis is a condition in which the size of the spinal canal is below normal, which may be congenital (congenital stenosis) or acquired (degenerative stenosis) [4]. Regardless of whether the stenosis affects one or several levels of a given section of the spine, lumbar stenosis occurs more often in patients with a congenital narrow spinal canal who have secondary degeneration of the spinal structures [5]. The peak incidence is recorded in the 5th and 6th decade of life [6,7]. Lumbar stenosis most often affects the L4/L5 level, then L3/L4, L2/L3, and the L5/S1 and L1/L2 spaces are less frequently affected [8]. The degenerations that occur concern articular surfaces, the ligamentum flavum, and intervertebral discs. Other causes resulting in narrowing of the spinal canal include tumors of the spinal canal, post-traumatic changes, instability and spondylolisthesis that occurred after previous surgical treatment [9].

A typical, characteristic symptom of degenerative stenosis of the L/S spine is the occurrence of neurogenic claudication, which is characterized by pain in the lower limbs, numbness, tingling and decreased muscle strength in the lower limbs [10,11]. An integral part of the life of patients with degenerative stenosis in the L/S section of the spine is pain at rest and at night, which intensifies, for example, when sneezing [12].

Patients suffering from degenerative stenosis of the L/S spine adopt an anthropoid position [11,13,14]. Pain is also reduced in the sitting and lying positions, in which the physiological lordosis of the L/S segment is reduced and the spinal canal opens [11,13,14].

Surgical treatment of degenerative stenosis of the L/S spine involves decompression of the neural elements. For this purpose, the most common procedure is a laminectomy, during which the vertebral arch and yellow ligamentum flavum are removed, which results in the widening of the lumen of the spinal canal of the L/S section of the spine [15,16].

The clinical manifestation of degenerative stenosis of the L/S spine also has a molecular basis [17]. Byvaltsev et al. [17], based on their analysis of the literature, indicated that spinal stenosis of the L/S section of the spine is most dependent on the occurrence of mutations or polymorphic variants for the following genes: isoform 1 of transforming growth factor beta (TGF- β1). transforming growth factor beta 1), bone morphogenetic protein 2 (BMP2), pro-alpha 1 chain of type I collagen (COL1A1), pro- alpha 1 chain of type I collagen, alpha 2 type I collagen (COL1A2, pt ro-alpha1 chains of type I collagen) and fibroblast growth factor receptor 3 (FGFR3) [17]. This analysis also highlighted the importance of TGFβ-dependent signaling pathways in the pathogenesis of degenerative stenosis of the L/S spine [17].

TGF- βand BMP bone morphogenetic proteins have the ability to bind to receptor II specific for each protein (TGF- β RI/II; BMPRI/II). The interaction between TGF- β and TGF- β RI leads to the activation of the pathway dependent on Sma and Mad related proteins (SMAD) proteins, in particular through the phosphorylation of SMAD2/3/4 proteins. In the next stage, SMAD proteins are then transported from the area the cytoplasm to the cell nucleus, where they exhibit activity typical of transcription factors [18–20]. In turn, the binding of the BMP protein to receptor leads to the phosphorylation of SMAD1/5/8/4 proteins; then the resulting complex is also transported from the cytoplasm to the cell nucleus, where they regulate the expression of other genes [18–20].

The cytokine that is responsible for the thickening of the ligamentum flavum, which leads to a reduction in the lumen of the spinal canal of the spine, is TGF-β1 [21]. Excessive expression of TGF-β1 has been reported in the degenerated yellow ligamentum flavum and chondrocytes [21,22]. However, there was no expression of TGF- β1 in the non-thickened ligamentum flavum, which indicates the significant importance of changes in the expression of this TGF- β isoforms in the later stages of ectopic ossification [21]. We should also not forget about the importance of microRNA molecules in the etiopathogenesis of degenerative stenosis of the spinal canal, including the L/S section of the spine [23,24].

The aim of this study was to determine changes in the expression pattern of TGF- β1–3 in the yellow ligamentum flavum collected from patients of the study group compared to samples obtained from participants of the control group.

## Material and methods

### Ethics consideration

A positive opinion was obtained from the local Bioethics Committee at the District Medical Chamber in Kraków (224/KBL/OIL/2022). All procedures were performed in accordance with the recommendations contained in the 2013 Helsinki Declaration, and patient data were pseudo-anonymized.

Moreover, written, voluntary consent to participate in the study was obtained from each patient qualified for the study group before joining the study. In turn, the issues of obtaining clinical material from participants of the control group (postmortem) are regulated by the provisions of the Act of July 1, 2005 on the collection, storage and transplantation of cells, tissues and organs (Journal of Laws of 2020, item 2134). Article 5 of this Act is based on the structure of the opt-out clause [25].

### Characteristics of patients in the study group

The study group included 60 patients (29 women − 48.33%; 31 men − 51.67%) aged 59.87 ± 2.13 years, who were qualified for neurosurgical procedure of extended fenestration and foraminotomy as well as partial phallectomy due to stenosis. Spinal canal of the L/S section of the spine, taking into account the inclusion and exclusion criteria presented in Table 1.

**Table 1.** Criteria for inclusion and exclusion from the study group.

Inclusion criteria	Exclusion criteria
Confirmation of degenerative stenosis of the L/S spine in imaging studies	Exclusion of degenerative stenosis of the L/S spine in imaging tests
Caucasian race	Race other than Caucasian
Age 18–80	Age under 18 or over 80
There are no serious contraindications to surgical treatment	The occurrence of serious contraindications to surgical treatment
Not taking anticoagulants or discontinuing them as recommended after additional consultation	No consent to the proposed surgical treatment
Lack of effectiveness of conservative treatment for at least 6 months	Taking anticoagulants or not being able to stop taking them
Consent to the proposed surgical treatment	Efficacy of conservative treatment
	Previous surgical treatment at the level of the lumbar-sacral spine

L/S, lumbar-sacral spine.

Spinal stenosis of the L/S section of the spine was diagnosed in patients based on the results of the interview and physical examination, including the results of MRI imaging performed in SE T1, SE T1 fluid-attenuated inversion recovery, FSE T2 and short tau inversion recovery sequences in transverse sections. and sagittal layers using 3 mm and 4 mm thick layers.

### Assessment of the intensity of pain in the L/S section of the spine in patients of the study group

**Figure 1.**
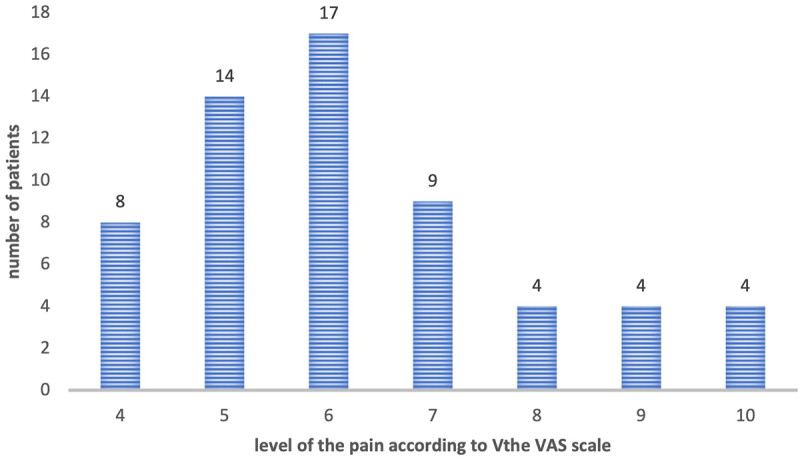
Number of patients in the study group declaring the intensity of pain in the L/S section of the spine at a given level according to the VAS scale.

The intensity of pain was assessed in each patient of the study group using a 10-point visual analogue scale (VAS), where 0 means no pain and 10 means very severe pain. Figure 1 shows the number of patients declaring pain at a specific level.

### Description of the neurosurgical procedure, including the postoperative period

General endotracheal anesthesia was used to perform the extended fenestration and foraminotomy procedure. In the first step, a skin incision was made over the space affected by the disease. Next, the paraspinal muscles were dissected, and then the hypertrophied yellow ligamentum flavum was removed using Kerrison bone biters. Then the opening was widened, a foraminotomy was performed and the hypertrophied joint fragments were removed, and the dural sac and nerve roots in the spinal canal were decompressed. The postoperative bed was rinsed with saline solution before the skin was sutured. Each procedure was performed with the assistance of an operating microscope.

If the patient did not experience complications in the early postoperative period, they were discharged from the neurosurgery department on the 3rd day after surgery with a recommendation to report to the Neurosurgical Outpatient Clinic 4 weeks after the procedure.

### Characteristics of control group participants

The control group consisted of 60 participants (28 women − 46.67% and 32 men − 53.33%), aged 49.17 ± 2.65 years), who were qualified to it based on the inclusion and exclusion criteria presented in Table 2. Control samples were obtained during forensic autopsy or organ donation. In order to confirm the lack of degenerative changes in the collected samples, Hematoxylin & Eosin (H&E) staining was performed for each of them, according to the manufacturer’s recommendations. For this purpose, two specialists in the field of neurosurgery (D.S. and R.S.) independently qualified yellow yellow ligamentum flavum for the control group.

**Table 2.** Inclusion and exclusion criteria for the control group.

Inclusion criteria	Exclusion criteria
Caucasian race	Race other than Caucasian
Age 18–80	Age under 18 or over 80
No current and/or past history of degenerative spine disease, no lifetime history of traumatic spine damage, especially in the lumbar-sacral spine	Current and/or past history of degenerative spine disease, lifetime history of traumatic spine damage, especially in the lumbar-sacral spine
No information about previous or current cancer	Information about previous or current cancer
Confirmation of the absence of degenerative stenosis of the L/S segment in H&E staining	Visualization of degenerative stenosis of the L/S segment in H&E staining

L/S, lumbosacral spine; H&E, hematoxylin & eosin staining.

The inclusion and exclusion criteria for the control group are presented in Table 2.

### Securing the collected material for molecular testing

Yellow ligaments collected from patients of the study group and participants of the control group, after being thoroughly washed from blood, were placed in a sterile Eppendorf tube to which the RNAlater reagent (Invitrogen Life Technologies, Carlsbad, CA, USA) was added and stored in low-temperature conditions (−80°C) until the molecular part of the experiment began.

### Determination of the TGF-β-1–3 mRNA expression profile in yellow ligamentum flavum samples collected from patients of the study group and participants of the control group

#### Total ribonucleic acid (RNA) extraction

Total RNA extraction from study and control samples was performed using the modified Chomczyński-Sacchi method using TRIzol reagent (Invitrogen Life Technologies, Carlsbad, CA, USA), according to the manufacturer’s recommendation.

In the first step, test and control samples were homogenized using a hand-held homogenizer (T18 Digital Ultra-Turrax, IKA Polska Sp. z o. o., Warsaw, Poland) until no solid fragments were visible. Then, 1 ml of TRIzol reagent was added to the Eppendorf tube and the whole was incubated for 5 minutes at room temperature, then 0.2 ml of chloroform (POL-AURA, Dywity, Poland) was added, after which the whole was mixed intensively and centrifuged. The upper phase containing RNA was collected and 0.5 ml of isopropyl alcohol (POL-AURA, Dywity, Poland) was added, and then the whole was incubated at −20°C for 12 hours. After this time, the whole thing was centrifuged and the precipitated RNA was washed twice with a 70% ethyl alcohol solution (POL-AURA, Dywity, Poland).

In order to remove possible contamination with genomic DNA, RNA isolates were purified using the DNAse I reagent (MBI Fermentas, Vilnius, Lithuania) and the RNeasy Mini Kit (Qiagen, Valencia, CA, USA), according to the protocol sent by the manufacturer.

After this procedure, the extracts were dried and stored in this form at −80°C until the next stages of molecular analysis began.

#### Qualitative and quantitative assessment of RNA extracts

First, dry RNA extracts were dissolved in 0.2 ml of deionized, RNase-free water, which allowed for qualitative and quantitative assessment of the obtained RNA isolates.

Qualitative evaluation of RNA extracts was performed by performing electrophoretic separation on a 1% agarose gel stained with 0.5 mg/ml ethidium bromide (SigmaAldrich, St. Louis, MO, USA). Obtaining two visible bands in the electrophoretic separation corresponding to the 28SrRNA and 18SrRNA fractions was considered a correct result.

In turn, the quantitative assessment of RNA extracts was performed using the spectrophotometric method. Deionized, RNase-free water was used to calibrate the device, the same water in which the RNA isolates were dissolved. The degree of purity of RNA extracts was assessed based on the absorbance ratio at 260 nm and 320 nm, which should be in the range of 1.80–2.00.

#### Real-time polymerase chain reaction preceded by reverse transcription RT-qPCR

The RT-qPCR reaction was performed in a 50 µL reaction mixture, and the thermal profile of the reaction was as follows: reverse transcription (45°C, 10 min), polymerase activation (95°C, 2 min) and 40 three-step cycles including denaturation (95°C, 5 s), hybridization (60°C, 10 s) and annealing (72°C, 5 s). Primers for the RT-qPCR reaction were purchased from Genomed (Poland), and their nucleotide sequence is given in Table 3. Glyceraldehyde-3-phosphoaldehyde dehydrogenase (GAPDH) was used as an endogenous control for the RT-qPCR reaction. Three technical replicates were performed for each biological replicate.

**Table 3.** Nucleotide sequence of primers used in the RT-qPCR reaction for TGF- β1–3 and GAPDH.

mRNA	Sequence oligonucleotide	Tm (°C)
*TGF- β1*	Forward: 5”-GGCCAGATCCTGTCCAAGC-3”	85.4
	Reverse 5”-GTGGGTTTCCACCATTAGCAC-3”	
*TGF- β2*	Forward: 5”-CAGCACACTCGATATGGACCA-3”	88.7
	Reverse 5”-CCTCGGGGCTCAGGATAGTCT-3”	
*TGF- β3*	Forward: 5”-CTGGATTGTGGTTCCATGCA-3”	86.6
	Reverse 5”-TCCCCGAATGCCTCACAT-3”	
*GAPDH*	Forward: 5”-GGTGAAGGTCGGAGTCAACGGA-3”	86.4
	Reverse 5”-GAGGGATCTCGCTCCTGGAAGA-3”	

Forward, sensible starter; Reverse, antisense primer; Tm, melting temperature; *GAPDH*, Dehydrogenase 3-phosphoglyceraldehyde; *TGF-* β*1–3*, transforming growth factor beta 1–3.

Changes in gene expression were presented as normalized relative mRNA expression (method 2 ^–∆∆Ct^), where 1 means equal expression of a given gene in the study and control samples; a score below 1 corresponds to a decrease in gene expression in the test samples compared to the control, and a score above 1 indicates overexpression of a given gene in the test samples compared to the control.

**Figure 2.**
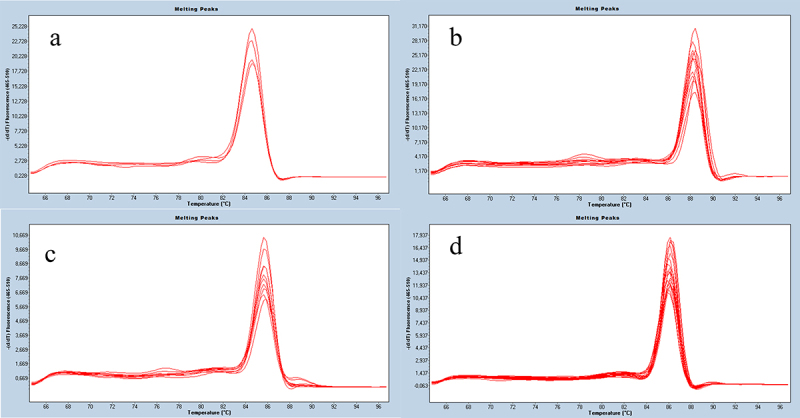
Examples of melting point curves of RT-qPCR products for TGF-β1 (A), TGF-β2 (B), TGF-β3 (C), and GAPDH (D).

The specificity of the RTqPCR reaction was confirmed by the melting temperature (Tm) of the PCR product. Tm for each amplimer, the value of which is given in Table 4, and the graphical interpretation of these data is presented in Figure 2.

**Table 4.** Concentration of TGF- β1–3 in the ligamentum flavum depending on the intensity of pain.

Isoform of TGF-β	VAS	Concentration TGF- β1 [pg/mg]	95%Cl	p (ANOVA test)
TGF- β1	4	1526.58 ± 196.29	1362.48–1690.68	0.51
	5	1679.41 ± 307.97	1493.30–1865.51	
	6	2773.02 ± 4267.83	498.85–5047.18	
	7	1742.20 ± 296.73	1529.94–1954.47	
	8	4392.17 ± 5330.23	0–12873.75	
	9	1637.10 ± 155.65	1443.83–1830.37	
	10	1692.64 ± 131.31	1483.68–1901.61	
TGF- β2	4	1987.19 ± 115.87	1545.19–243.12	0.79
	5	1876.98 ± 198.98	1243.10–2456.17	
	6	2876.198 ± 234.54	2176.14–3451.19	
	7	2256.12 ± 198.18	1765.11–3091.18	
	8	1876.19 ± 245.19	1567.19–2341.81	
	9	1987.23 ± 145.16	1541.19–1908.18	
	10	2098.18 ± 176.12	1762.12–2198.12	
TGF- β3	4	416.98 ± 21.22	201.18–541.12	0.63
	5	456.78 ± 16.78	398.,19–487.19	
	6	514.18 ± 19.12	402.11–601.12	
	7	402.17 ± 10.73	367.13–478.98	
	8	578.11 ± 76.15	456.71–702.34	
	9	521.91 ± 65.18	398.19–578.24	
	10	402.12 ± 20.14	323.76–476.54	

TGF- β1–3, transforming growth factor beta 1–3; p, value of statistical significance; mean±standard deviation; 95%Cl, 95% confidence interval; 95% confidence interval; VAS, visual analogue scale. Data were shown as mean standard deviations.

### Test enzyme-linked immunosorbent assay (ELISA)

#### Samples preparation

Samples of yellow ligamentum flavum collected from patients in the study group and participants in the control group, minced using a scalpel, were transferred to a new Eppendorf tube and weighed. The prepared samples were incubated with a solution of 4 M guanidine hydrochloride (Sigma Aldrich St. Louis, MO, USA), 1 M sodium acetate (Sigma Aldrich St. Louis, MO, USA), Triton 2% (Sigma Aldrich St. Louis, MO, USA). and protease inhibitor cocktail (Sigma Aldrich St. Louis, MO, USA) for 12 hours at 4°C on a laboratory rocker. Next, the samples were centrifuged, and the obtained supernatant was stored at −20°C until the next stage began.

#### Antibodies used in the ELISA test

The following antibodies were used for the ELISA test: polyclonal anti-TGF-β1 antibody bs-0086 R (STI, Poznań, Poland); polyclonal anti-TGF-β2 antibody bs -20,412 R (STI, Poznań, Poland), polyclonal anti-TGF-β3 antibody bs-0099 R (STI, Poznań, Poland) and polyclonal anti-GAPDH antibody (ThermoFisher Scientific, Waltham, MA, USA) according to with the manufacturer’s recommendation.

#### ELISA test procedure

Antibodies used in the ELISA were diluted in phosphate-buffered saline (PBS; pH 7.6; Merck, Sigma Aldrich, St. Louis, MO, USA) and then spotted on Pierce NeutrAvidin plates (Thermo Fisher Scientific, Waltham, MA, USA). The whole thing was incubated at room temperature for 120 minutes. After this time, the plates were washed three times with blocking solution (1% bovine serum albumin [BSA] in PBS; Sigma Aldrich, St. Louis, MO, USA). Then, 50 μL of standards or samples diluted 1:500 were added to individual wells and incubated for 180 minutes at room temperature. After this time, the wells were washed three times with PBS solution. After this step, specific anti-TGF-β1–3 antibodies conjugated with HRP were added and the whole was incubated again for 180 minutes at room temperature. In the last step, each plate was washed three times with phosphate buffer and 100 μl of BM Chemiluminescence ELISA Substrate (buffered solution containing luminol/4-iodophenol and buffered solution containing a stabilized form of H_2_O_2_ ; Sigma Aldrich, St. Louis, MO, USA) was added. An M200PRO plate reader (Tecan, Männedorf, Switzerland) was used to evaluate absorbance at 540 nm.

Samples that did not contain primary anti-TGF-β1–3 antibodies were negative controls. However, the human cervical cancer cell line HeLa was used as a positive control. All samples were analyzed in triplicate. Average values were used for analysis.

### Western blot

#### Sample preparation

In the first stage, samples of yellow ligamentum flavum collected from patients of the study group and participants of the control group were rinsed with a PBS solution and then placed in a new Eppendorf tube to which 0.50 ml of radioimmunoprecipitation assay buffer (RIPA; Sigma Aldrich St. Louis, MO, USA) supplemented with a cocktail of protease and phosphatase inhibitors (Sigma Aldrich St. Louis, MO, USA). The samples were then homogenized using a hand-held homogenizer (T18 Digital Ultra-Turrax, IKA Polska Sp. z o. o., Warsaw, Poland) until no solid fragments were visible. After this step, the tubes were placed on ice and gently mixed on a rocker for 60 minutes, after which the samples were centrifuged and the supernatant was collected and stored at −80°C until further analysis.

After thawing, the total protein concentration in the preparations was determined using the KIT bicinchoninic acid assay (BCA; Thermo Fisher, Waltham, MA, USA) according to the manufacturer’s guidelines. Protein concentrations ranged from 20 to 100 μg of total protein. All protein concentration measurements were calculated using a standard curve based on standard solutions of bovine serum albumin (BSA; including a set of six standard points − 0, 250, 500, 1000, 1500, and 2000 µg/ml) (Sigma Aldrich St. Louis, MO, USA).

#### Antibodies used in the Western blot test

The same primary anti-TGF- β1–3 antibodies were used for Western blot analysis as in the ELISA test, but diluted in a ratio of 1:1000. HRP-conjugated goat anti-rabbit IgG (BioRad, Milan, Italy) dilution 1:3000 was used as the secondary antibody. GAPDH (Santa Cruz Biotech, Dallas, Texas, USA; dilution 1:500) was used as an endogenous control.

#### Western blot procedure

An equal amount of protein (20 µg) was introduced into each well of a sodium dodecyl sulfate polyacrylamide gel (POL-AURA, Dywity, Poland) and electrophoretic separation was performed. The next step in the analysis was to transfer the proteins to a polyvinylidene membrane (PVDF, pore size 0.45 µm, Thermo Fisher, Waltham, MA, USA), which was then blocked (1X Tris-buffered saline (TBS) containing 0.1% Tween-20 (Sigma Aldrich St. Louis, MO, USA) supplemented with 5% nonfat dry milk (Merck, Sigma Aldrich St. Louis, MO, USA). After this step, anti-TGF-β1–3 primary antibody was added and incubating for 12 hours at + 4°C). The next day, the membrane was washed, and the secondary antibody was added. The optical density of each product was measured using Kodak MI 4.5SE software (KODAK, Rochester, NY, USA).

Samples that did not contain primary anti-TGF-β1–3 antibodies were negative controls. However, the human cervical cancer cell line HeLa was used as a positive control.

#### Immunohistochemical (IHC) analysis

Tissue specimens were sliced at a thickness of 8.0 µm using a microtome (Leica Microsystems, Germany). Subsequent processing steps, including dehydration, antigen retrieval, antibody incubations, and staining, were carried out in accordance with the manufacturer’s guidelines provided in the instruction manuals for the DAB Substrate Kit (Peroxidase, HRP; from Vector Laboratories, Newark, California, USA), and the IHC-Paraffin Protocol (IHC-P; from Abcam plc, Cambridge, UK).

The resulting immunohistochemical reactions were observed and captured using a Nikon Coolpix fluorescent optical system. The cellular localization and quantity of the selected proteins were analyzed through computer image analysis utilizing the ImageJ software. A total of 15 photographs were taken from three slides per patient under 200× magnification.

Using the ImageJ software, specifically the IHC-Profiler plug-in, the optical density of DAB reaction products was assessed in the areas where the immunohistochemical reaction occurred in reaction to the presence of the selected proteins. Additionally, the average percentage of the DAB-stained area was calculated relative to the background values in each field.

#### Statistical analysis

Statistical analysis was performed in the Statplus program (AnalystSoft Inc, Brandon, FL 33,511, USA), and the statistical significance threshold (p) < 0.05 was adopted to verify the hypotheses. The first stage of the statistical analysis was to perform the Shapiro-Wilk test to determine whether the distribution of the obtained data was consistent with the assumptions of normal distribution. Based on the obtained result, there were no grounds to reject the null hypothesis and accept the alternative hypothesis, therefore the next stages of statistical analysis were carried out using parametric tests, i.e. one-way ANOVA or Student’s t-test. In a situation where the ANOVA analysis indicated the occurrence of statistical significance, Scheffe’s post-hoc test was performed to determine which comparisons the differences were significant.

## Results

### TGF-β-1–3 mRNA expression profile determined using RTqPCR

**Figure 3.**
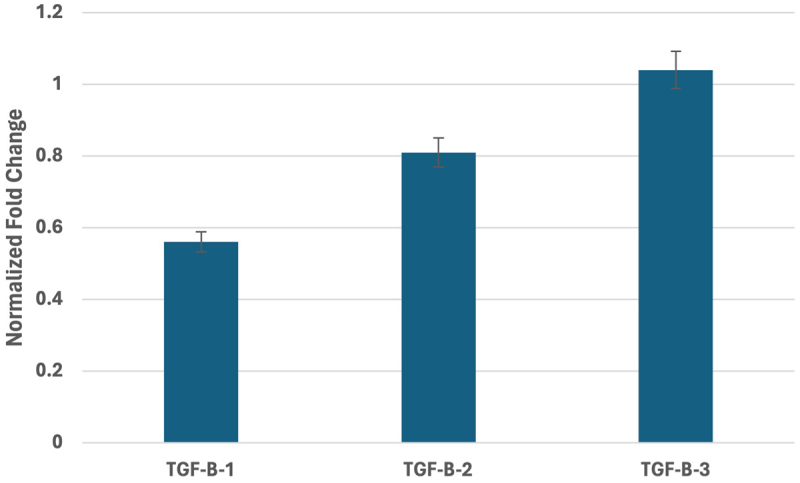
TGF-β-1–3 expression profile in the yellow ligamentum flavum collected from patients of the study group compared to samples obtained from participants of the control group. TGF-b-1–3, transforming growth factor beta 1–3. Data were shown as mean ±standard deviations.

At the mRNA level, silencing of *TGF-β-1* expression by 56.1% compared to the control was observed in samples taken from patients of the study group. Silencing of expression was also noted for *TGF-β-3* in samples taken from patients of the study group

by 18.70% compared to the transcript expression in the study samples. However, for *TGF-β-2*, expression in the study samples increased by only 4% compared to the control. Figure 3 shows _ standardized relative *TGF-β-1–3* mRNA expression .

### TGF-β-1–3 expression β in the yellow ligamentum flavum collected from patients of the study group and participants of the control group determined using the ELISA test

**Figure 4.**
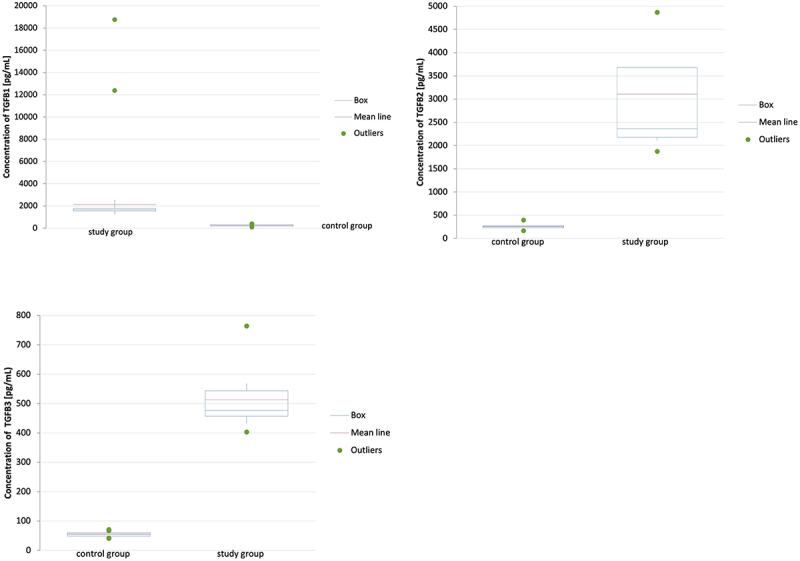
Concentration of TGF- β1–3 in the yellow ligamentum flavum collected from patients of the study group and participants of the control group obtained by ELISA test. TGF- β1–3, transforming growth factor beta 1–3; *, statistically significant difference between groups (Student’s t-test; *p* < 0.05). Data were shown as mean ±standard deviations

At the protein level, a statistically significant higher concentration of TGF-β1 was recorded in the yellow ligamentum flavum obtained from patients of the study group and participants of the control group (2139.33 pg/mL ±2593.72 pg/mL vs. 252.45 pg/mL ±83.89 pg/mL; *p* < 0.0001; Figure 4).

In samples of the yellow ligamentum flavum collected from patients of the study group, the concentration of TGF-β2 at the protein level was significantly higher than in control samples obtained from participants of the control group (3104.34 pg/mL ±1192.74 pg/mL vs. 258.86 pg/mL ± 82.98; *p* < 0.0001; Figure 4).

In samples of the yellow ligamentum flavum taken from patients of the study group, the concentration of TGF-β3 at the protein level was significantly higher than in control samples obtained from participants of the control group (512.75 pg/mL ±107.36 pg/mL vs. 55.06 pg/mL ±9.83 pg/mL; p < 0.0001; Figure 4).

### TGF-β-1–3 expression in the yellow ligamentum flavum depending on the severity of pain reported by patients

Then, changes in TGF- β-1 concentration were assessed in samples of the yellow ligamentum flavum taken during neurosurgery from patients of the study group depending on the degree of declared pain intensity. Nevertheless, one-way ANOVA showed no statistically significant differences between TGF-β-1 concentration and the intensity of pain declared by patients (Table 4; *p* > 0.05). Also, one-way ANOVA showed no statistically significant differences between TGF-β-2 concentrations and the intensity of pain declared by patients (Table 4; *p* > 0.05). Then, changes in TGF-β-3 concentration were assessed in samples of the yellow ligamentum flavum taken during neurosurgery from patients of the study group depending on the degree of declared pain intensity. Nevertheless, one-way ANOVA showed no statistically significant differences between TGF-β-3 concentrations and the intensity of pain declared by patients (Table 4; *p* > 0.05).

### Expression of TGF-β-1–3 proteins determined by Western blot

**Figure 5.**
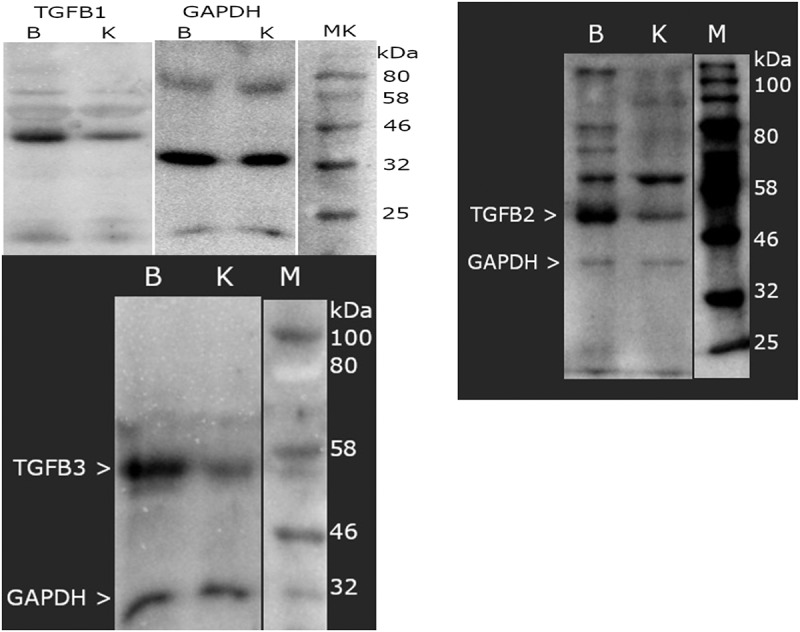
Example of electrophoretic separation of TGF- β1–3 and GAPDH in study and control samples. TGF β-1–3, transforming growth factor beta 1–3; GAPDH, glyceraldehyde-3-phosphate dehydrogenase; kDa, kilo Daltons; M, size marker (New England Biolabs Marker); B, sample of yellow ligamentum flavum taken from the study group; K, yellow ligamentum flavum sample from control group participants.

Figure 5 shows an example electropherogram that confirms the specificity of the reaction (based on the molecular weight of TGF- β-1 and obtaining a band with a molecular weight of 44 kDa in Chapter 1; based on the molecular weight of TGF-β2 and obtaining a band with a molecular weight of 50 kDa in Chapter 1; based on the molecular weight of TGF- β3 and obtaining a band with a molecular weight of 47 kDa in Chapter 1) and the naiveness of the samples (based on the GAPDH result; molecular weight 37 kDa).

TGF-β-1 normalized to GAPDH was 11.13 ± 8.50 in the study samples and 0.95 ± 1.36 in the control samples (*p* < 0.05). The optical density of bands for TGF-β-2 normalized to GAPDH was 5.90 ± 3.28 in the study samples and 0.75 ± 0.68 in the control samples (*p* < 0.05). In turn, TGF-β-3 normalized to GAPDH was 1.58 ± 2.29 in the study samples and 0.15 ± 0.07 in the control samples (*p* < 0.05).

### TGF-β-1–3 expression profile determined by IHC

**Figure 6.**
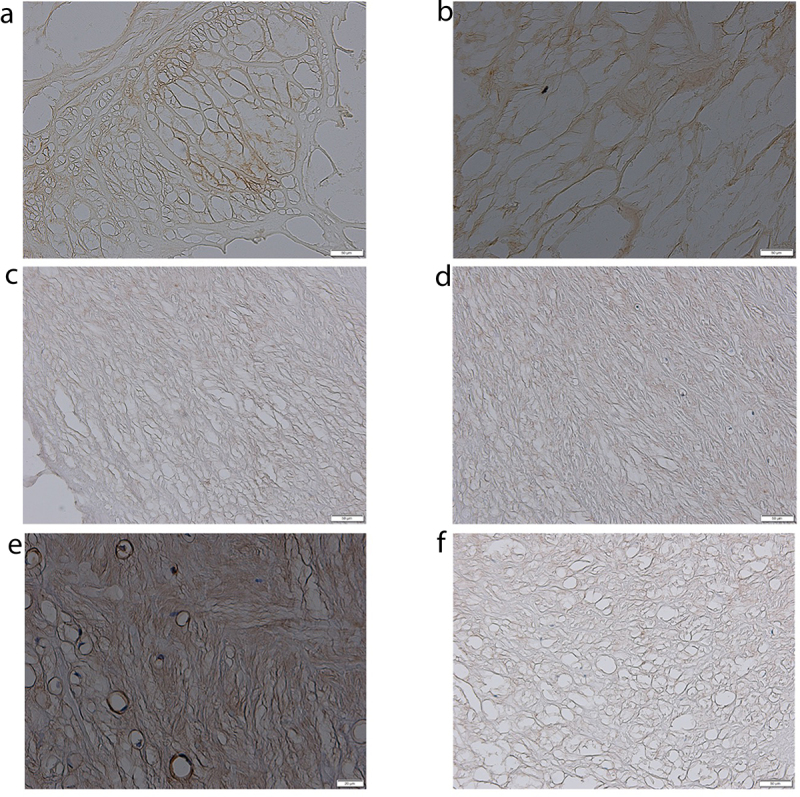
Immunochemical expression of TGF-β −1–3 in the study and control samples. TGF-β −1–3, transforming growth factor beta 1–3; A – expression of TGF-β-1 in the control group; B – expression of TGF-β-1 in the study group; C – expression of TGF-β-2 in the control group; D – expression of TGF-β-2 in the study group; E – expression of TGF-β-3 in the control group; F – expression of TGF-β-3 in the study group.

In both degenerated and control ligaments flavum, a colored IHC reaction product was visible for all three TGF-β-1–3 isoforms (Figure 6). The optical density of the reaction product for TGF-β-1 in the degenerated yellow ligamentum flavum reached 344.27% of control (Table 5; *p* < 0.05). The optical density of the reaction product for TGF-β-2 and TGF-β-3 reached 231.25% and 176.19% of the control, respectively (Table 5; *p* < 0.05). In addition, Figure 6 shows example images of IHC analysis for the TGF-β-1–3.

**Table 5.** Optical density of the reaction product for selected proteins in ligamentum flavum obtained from the study and control groups.

Proteins	Degenerative ligamentum flavum	Control ligamentum flavum	p-value
TGF-β −1	10.81 ± 1.75	3.14 ± 0.98	*p* < 0.0001
TGF-β −2	2.59 ± 0.65	1.12 ± 0.28	p = 0.002
TGF-β −3	2.22 ± 0.80	1.26 ± 0.31	p = 0.001

TGF-β −1–3, transforming growth factor beta 1–3. Data were shown as mean ±standard deviations.

## Discussion

The incidence of degenerative spine disease, especially the L/S section, is constantly increasing, which is influenced by changes in the demographic structure in society, a sedentary lifestyle, reduced physical activity, and changes in eating habits, which indirectly contribute to increasing body weight and thus strength. acting on the spine [26–29].

The factors that significantly contribute to the occurrence of the degenerative process in the spine include genetic predispositions, the degree of muscle tension, chronic mechanical stress and the level of neuromuscular reactivity [30,31].

The degenerative process of the spine is a chronic, slowly progressing condition, characterized by the progression of existing changes, which is associated with the induction and development of inflammation [32,33]. Inflammatory processes and the associated release of cytokines may contribute to the development and progression of degenerative spine disease [32,33]. Cytokines are a broad group of small signaling proteins that play a key role in cellular signaling and communication [28]. They are secreted by various cells of the immune system as well as by other cell types such as epithelial cells, chondrocytes, osteoclasts, fibroblasts and endothelial cells [28]. Cytokines act as molecular messengers, transmitting signals between cells and regulating immune responses, inflammation, and various other biological processes [28,34]. They are involved in both pro-inflammatory and anti-inflammatory responses and play an important role in maintaining immune homeostasis [28,34]. Cytokines can regulate cell growth, differentiation, migration and activation, and mediate communication between immune cells and other cells in the body [28,34]. Moreover, these molecules perform various functions and may act in an autocrine, paracrine or hormonal manner [28,34]. Cytokines include interleukins (such as IL-1, IL-2, IL-6 and IL-10), interferons, tumor necrosis factors, TGF-βs, chemokines and many others [28,34].

In the course of degenerative spine disease, a reduced inflow of nutrients to cartilage structures is observed, which translates negatively into the biosynthesis of collagen fibers and ECM degradation as a result of overexpression of, among others, matrix metalloproteinases (MMPs) [35,36]. As a consequence, there is an increase in the concentration of pro-inflammatory cytokines locally, the source of which are macrophages arriving at the site of the inflammatory process, as well as degenerated cartilage tissue [35,36].

At the molecular level, it is indicated that the degenerative process in the yellow ligamentum flavum is primarily responsible for a change in the TGF-β-1–3 concentration profile and the resulting abnormalities in cell signaling dependent on the above-mentioned cytokine [37–40].

Studies conducted by Cao et al. [41] and Amudong et al. [42] confirmed that TGF- β-1 is one of the most essential cytokines that determines the hypertrophy and degeneration of the yellow ligamentum flavum at the molecular level [41,42]. Moreover, Cao et al. [41] the significant importance of TGF- β in the induction of degenerative spinal stenosis to the fact that the above-mentioned cytokine induces the proliferation of fibroblasts and osteoblasts, the biosynthesis of MMP proteins, which results in narrowing of the spinal canal lumen [41]. It should be remembered that under the influence of TGF- β, fibroblasts differentiate into myofibroblasts, which are characterized by a higher secretion of extracellular matrix proteins than fibroblasts [43]. The key importance of TGF-β in the induction and development of degenerative spinal stenosis is also related to the fact that this cytokine is an inhibitor of MMP expression and an activator of MMP inhibitors [44,45].

Nevertheless, based on the analyzed literature, it seems that TGF-β-1 is the most important isoform of the discussed cytokine associated with the degenerative process within the yellow ligamentum flavum [46]. Burt et al. [46] found in an animal model of degenerative spinal stenosis an increase in TGF-β-1 expression in the ligaments flavum of the study group compared to the control [46], which is consistent with the pattern of TGF-β-1 expression at the protein level, which was noted in my work [46]. Therefore, it appears that TGF-β-1 is the dominant factor contributing to the remodeling of the ligamentum flavum, which involves replacing elastin fibers with collagen fibers [47–49]. Also, Yabu et al. [50] noted an increase in the expression of TGF-β 1, IL-6, α –smooth muscle actin and collagen type 1 alpha in the hypertrophied ligaments flavum of the L/S spine, indicating the significant involvement of signaling pathways dependent on from IL-6 [50].

β in degenerative spine disease is indicated. However, this effect lasts only up to a certain level (which has not yet been precisely determined), as exceeding it induces degenerative changes in the intervertebral disc [51,52]. The different biological effects exerted by TGF-β may therefore depend on the etiology of the degenerative spine disease, i.e. intervertebral disc degeneration or yellow ligamentum flavum hypertrophy, as well as the activated TGF-β signaling pathways [51,53–56].

Therefore, in this article, changes in the TGF-β1–3 concentration profile at the mRNA and protein levels in degenerated ligaments flavum were assessed, as well as whether their expression profile correlated with the intensity of pain reported by patients of the study group.

The yellow ligamentum flavum was used as a comparative material, which was collected postmortem and preserved for further stages of molecular research using the RNA later reagent, which, according to the manufacturer’s recommendation, has protective properties, allowing for determinations at the transcriptome and proteome level [57,58]. It should also be noted that if we want to use clinical material of human origin in research on degenerative spine disease, there is no other way to obtain it than postmortem sampling. Research conducted by other research teams also indicates that the material was collected from participants postmortem is fully valuable in order to be able to compare the obtained results, e.g. the concentrations of the assessed factors in tissues affected by the degenerative process [59,60].

Based on the research conducted as part of our research, a different expression profile of TGF-β1–3 was found at the mRNA and protein levels. For *TGF-β-1* mRNA and *TGF-β-3* mRNA , their expression was silenced in the study samples compared to the control, while the expression of *TGF-β-2* mRNA was at a similar level as in the control samples. However, at the protein level, higher concentrations of all three TGF-β isoforms were found in the yellow ligamentum flavum collected from patients during neurosurgical surgery compared to samples obtained from control group participants (*p* < 0.05). The observed different pattern of *TGF-β-1–3* expression at the mRNA and protein levels may have several reasons.

First of all, it should be remembered that the processes of transcription and translation do not occur simultaneously in cells and occur in two separate compartments [61,62]. Transcription is the first stage of gene expression in which the deoxyribonucleic acid (DNA) sequence is used as a template for the synthesis of a complementary messenger RNA (mRNA) molecule [61,62]. Transcription takes place in the nucleus of a eukaryotic cell and involves the enzyme RNAII polymerase and transcription factors [61,62]. The resulting transcript, which is complementary to the template DNA strand and homologous to the coding strand in eukaryotic cells, undergoes a maturation process that includes the addition of a guanyl cap at the 5' end of the mRNA, the addition of a poly-A tail at the 3' end of the mRNA, and splicing [61–63].

After transcription, the mature mRNA molecule is transported from the cell nucleus to the cytoplasm, where translation takes place, i.e. the process of protein synthesis based on the information encoded in the mRNA [61,62]. During translation, the ribosome reads the mRNA codons and matches them with the appropriate transfer RNA (tRNA) molecules carrying amino acids [29,64,65]. Amino acids are linked together to form a polypeptide chain that folds into a functional protein [29,64,65].

It is important to remember that although transcription and translation are separate processes, they are interrelated [29,64,65]. The mRNA molecule produced during transcription serves as a template for protein synthesis during translation [29,64,65]. Once transcribed, mRNA can repeat many times subject process translation [44,45], which may partially explain the results I obtained.

The second potential mechanism that explains the obtained results is related to the degradation of TGF-β-1–3 *mRNA*, e.g. by micro RNA molecules (miRNA), which, showing incomplete complementarity to the target transcript, reduces the number of mRNA molecules that will be subject to translation. MiRNAs are small, single-stranded RNA molecules that play a key role in the post-transcriptional regulation of gene expression [66,67]. They are a class of non-coding RNA molecules, which means that they do not encode proteins themselves but instead perform regulatory functions [66,67]. They have microRNAs usually length approximately 21 to 25 nucleotides [66,67]. Mature miRNAs bind to specific mRNA molecules, usually in the 3’ untranslated region (UTR), through partial complementary base pairing [66,67]. This interaction between miRNA and mRNA can lead to mRNA degradation or translation inhibition [66,67].

Many studies published so far have shown that both TGF-β-1–3 expression can be regulated by miRNA molecules and that the cytokine itself causes changes in the miRNA concentration profile [68,68,69,69–75]. Davis et al. [73,74] showed in two studies that SMAD proteins directly influence the biogenesis of miRNA and that TGF- βand BMP, acting synergistically, increase the expression of miR-21, as well as modulate the formation of the active form of this miRNA [73,74]. The regulation of miRNA expression and biogenesis by ligands such as TGF-β has also been confirmed in relation to the p53 protein, called the “guardian of the genome” [75,76], p53]. TGF-β contributes to the overexpression of miR-21, mir-181, miR-10b, mir-17, miR-92, miR-155, mir-192, miR-23, miR-24, miR-27, miR-216, miR- 217, miR-494, miR-182 [68,77–79] and silencing the expression of miRNAs such as miR-200, miR-203, let-7, miR-34a, miR-584 [68–72,77]. Moreover, the existence of mutual dependencies between the regulation of TGF-β concentration and TGF-β-dependent signaling pathways by miRNAs and vice versa has been demonstrated. For example, the miR-200 family has the ability to interact with mRNAs encoding SMAD2 and TGF-β receptor I (TGF-βRI). Therefore, downregulation of miR-200 expression by TGF-β results in activation of SMAD-dependent signaling pathways [80,81]. In addition, recent studies confirm that the miRNA expression profile changes during the degenerative process in the yellow ligamentum flavum [82–84], which undergoes hypertrophy, narrowing the lumen of the spinal canal, and consequently causing pain in patients.

Third, proteins encoded by *TGF-β-1–3* mRNA may have an extended half-life, which allows them to accumulate in the cell even when expression at the mRNA level is silenced. However, based on the analysis of the available literature, there is no evidence that would confirm this hypothesis [85]. Only Wakafield et al. [85]showed in their study that TGF-β-1 has a very short half-life in the plasma of rats, and this half-life can be extended through appropriate modifications, including the use of βthe LAP-related TGF-1 [85].

However, in the current study, we did not demonstrate a relationship between the expression profile of TGF-β-1–3 in the yellow ligamentum flavum and the degree of pain assessed according to the VAS scale, which the participants declared. This may be due to the fact that the VAS scale is a subjective tool, so the level of pain experienced is actually an individual feature, and the failure to demonstrate the discussed relationship may be partly due to the disproportion in the number of participants declaring a given level of pain intensity [86–88].

Despite the comprehensive examination of the expression profile of TGF-β1–3 at both the mRNA and protein levels within the yellow ligamentum flavum, this study has several limitations that warrant acknowledgment. Firstly, the sample size in our study might not have been sufficient to capture the full spectrum of variability within the population, potentially limiting the generalizability of our findings. Moreover, the subjective nature of pain assessment using the VAS could have introduced variability in reported pain intensity, impacting the correlation analysis with TGF-β expression levels. Furthermore, while we attempted to control for confounding variables, other factors influencing TGF-β expression and signaling pathways may not have been fully accounted for in our analysis. Finally, the study’s cross-sectional design limits our ability to establish causality or temporal relationships between TGF-β expression and degenerative spine disease progression. Future research with larger sample sizes, longitudinal designs, and more comprehensive control of potential confounders is needed to address these limitations and further elucidate the role of TGF-β in degenerative spine disease.

Summarizing, taking into account the results of the expression pattern of TGF-β-1–3 at the mRNA and protein levels obtained in this dissertation, together with data from the literature, it can be concluded that TGF-β-1 plays a key role in the hypertrophy of the ligamentum flavum, which leads to stenosis of the L/S segment. spine. However, the role of TGF-β and the pathways dependent on this cytokine in the context of degenerative spine disease is different, i.e. when the source of pain is degeneration of the intervertebral disc, TGF-β rather plays a protective role, and in the case of degenerative spinal stenosis, TGF-β should be seen as a factor strengthening the degenerative process of the ligamentum flavum. It was also confirmed that the degenerative process accompanying degenerative stenosis of the L/S spine is pro-inflammatory, so modern forms of therapy should focus on restoring the normal concentration of TGF-β and activated signaling pathways, as well as taking into account the regulatory role of miRNAs in controlling TGF-β concentration [89,90].

## Data Availability

All data was included in the paper.
